# Context/Resource-Aware Mission Planning Based on BNs and Concurrent MDPs for Autonomous UAVs

**DOI:** 10.3390/s18124266

**Published:** 2018-12-04

**Authors:** Chabha Hireche, Catherine Dezan, Stéphane Mocanu, Dominique Heller, Jean-Philippe Diguet

**Affiliations:** 1 Lab-STICC, CNRS, Université de Bretagne Occidentale, 29200 Brest France, France; 2 CNRS, Grenoble-INP, Inria, LIG, University of Grenoble-Alpes, 38000 Grenoble, France; stephane.mocanu@imag.fr; 3 Lab-STICC, CNRS, Université de Bretagne Sud, 56100 Lorient, France; dominique.heller@univ-ubs.fr (D.H.); jean-philippe.diguet@univ-ubs.fr (J.-P.D.)

**Keywords:** fault recovery, anomaly detection, diagnosis, mission planning, Markov Decision Process, Bayesian Networks, System-on-Chip

## Abstract

This paper presents a scalable approach to model uncertainties within a UAV (Unmanned Aerial Vehicle) embedded mission manager. It proposes a concurrent version of BFM models, which are Bayesian Networks built from FMEA (Failure Mode and Effects Analysis) and used by MDPs (Markov Decision Processes). The models can separately handle different applications during the mission; they consider the context of the mission including external constraints (luminosity, climate, etc.), the health of the UAV (Energy, Sensor) as well as the computing resource availability including CPU (Central Processing Unit) load, FPGA (Field Programmable Gate Array) use and timing performances. The proposed solution integrates the constraints into a mission specification by means of FMEA tables in order to facilitate their specifications by non-experts. Decision-making processes are elaborated following a “just enough” quality management by automatically providing adequate implementation of the embedded applications in order to achieve the mission goals, in the context given by the sensors and the on-board monitors. We illustrate the concurrent BFM approach with a case study of a typical tracking UAV mission. This case also considers a FPGA-SoC (FPGA-System on Chip) platform into consideration and demonstrates the benefits to tune the quality of the embedded applications according to the environmental context.

## 1. Introduction

The expected rise of autonomous vehicles will require a significant improvement of embedded decision-making capabilities. These decisions must consider the goal of the mission and adapt the choice of actions to achieve it according to random events related to the mission context, to the health of the whole system and dealing with the risk and the safety of the mission. Autonomy means also high performance computing with limited processing, communications and storage resources. This aspect is usually underestimated by roboticists, but it is crucial that decision-making includes this constraint to avoid any choice that would lead to failure because of insufficient computing resources.

In this work, we address the implementation of autonomous embedded intelligence to face uncertainty. We propose a new approach called concurrent BFM that combines Bayesian Networks (BN) built from Failure Mode and Effects Analysis (FMEA) to feed Markov Decision Process (MDP) with transitions’ probabilities. This approach is divided into two levels. The diagnosis level is based on BN and addresses resources availability, application quality of service (QoS) as well as system and sensor health status. The decision level is based on MDP; it is resource-aware, thus the application version can be adapted according to the resource availability and the mission policy.

Thanks to the modularity of the BN and the MDP models, the BFM model offers on the one hand an easy way to describe the mission by the user. On the other hand, it gives the possibility to separately describe different applications using concurrent MDPs. These applications can be active or not, and they can have different real-time constraints according to the mission phases. This modular approach of mission specification means scalability. It induces more MDPs but reduces the complexity of each MDP in terms of states and actions number. It means that the execution time of each MDP is decreased and then the response time of the decision module. It is also important to notice that the different MDPs can be executed in parallel when necessary. The whole BFM approach is also meant to be scalable from the designer point of view. Designers can actually specify an embedded mission planning to face uncertainty according to FMEA rules and decision policy. We demonstrate the approach with a real-life case study on tracking scenario. In addition, the BFM model offers the possibility to add as many MDPs as necessary to describe the mission and to define several BNs to evaluate the health status of the different system components.

The contributions of this paper are related to the following benefits of the BFM model:

- the BN modules are used to compute the probabilities associated with the transitions of the MDP model; it provides a more accurate description of the decision-making process.
- the BN modules can take into account different kinds of constraints including the use of computing resources, the availability of sensors and the application quality of service.
- the BFM model offers the possibility to describe a global mission with different styles according to designer choices. On one extreme, it can be monolithic, but the concurrent approach is more tractable. This ability of describing the system in a concurrent manner also enables reducing the response time of the decision-making process.

In Section 2, we present the state of the art and the motivations for this work. In Section 3, we introduce the decision model based on MDP. Then, we detail the BFM model in Section 4. Section 5 describes a complete mission using this model. In Section 6, we propose a concurrent version of the BFM model. Results and validation are shown in Section 7 and finally we provide conclusions.

## 2. Related Work

With the tremendous expansion of UAV applications, the number of publications related to the field exploded during the last decade. Topics covers a large spectrum of UAV related issues including flight control, optimal mission planning, cooperation and task assignment for UAV swarms, security, communication networks, target tracking, sensors reconfiguration and diagnostics and many more.

Specifically, our work concerns the health-aware mission planning of a single UAV. Mission planning has been an extensively studied problem since the beginning of the century (see [1], for example, as an early reference). Both stochastic and deterministic approaches were developed. Deterministic approaches are generally energy-optimization oriented and do not explicitly take into account uncertainties (see [2] or [3] for a recent reference), but they can provide a real-time mission controller. On the other hand, probabilistic approaches are able to handle environment and UAV health status uncertainties but will often need important computing resources to achieve online calculations.

With the autonomy of UAV, the security becomes a major issue to achieve the mission correctly. In the case of aerospace systems, a set of faults and errors [4] related to sensors, actuators and embedded systems can be identified in advance. To detect and to mitigate the different kinds of hazards, a number of model-based FDIR (fault detection isolation recovery) techniques have been introduced [5].

Environment uncertainties modelling was the subject of numerous works. A very complete and interesting survey may be found in [6]. Although the survey field is the spacecraft modelling and mission planning, the models are basically the same for the UAV mission planning.

The mission planning enables making decisions about the most appropriate actions to execute. The reconfiguration and the adaptation should be addressed online to face the unexpected hazards in a case of autonomous systems. Among the techniques available for decision-making, three approaches have emerged in the literature [7]: (1) Multicriteria Decision Making techniques, (2) Mathematical Programming techniques and (3) Artificial Intelligence. Nevertheless, to deal with uncertainty of the environment and of the system (external or internal hazards), fuzzy techniques [8] or stochastic/probabilistic models such as Bayesian Networks [9] are exploited. For an online adaptation/reconfiguration, we need to consider an efficient decision-making engine able to give the appropriate actions under time constraints, system constraints and environmental constraints. Typical models used for decision are based on MDP but genetic algorithms (GA), heuristics or mixed approaches also exist [10]. Those approaches (MDP or GA + heuritics) result in an important complexity (Worst-Case Execution Time in [10] approaches 500 ms). Our concurrent partial MDP approach is between 50 and 100 times faster than the best WCET in [10].

Between various mission planning applications, one can distinguish “mission oriented” approaches and “UAV preservation” approaches. Mission oriented applications will focus on the optimal achievement of the mission often assuming a higher risk of losing a drone while achieving the mission. This corresponds to military applications and usually assumes a cooperative swarm of UAVs collaborating for the mission achievement. The decision model might be Markov Decision Process oriented [11] or multi-objective evolutionary algorithms based [12,13]. UAV health oriented approaches will tend to avoid physical damage or loss of the drone. They correspond mainly to civilian applications like forest or agricultural surveillance drones and are tackling mostly single-drone cases. Our study falls in this category. We are concerned with the optimal online mission reconfiguration with respect to the drone health and environment uncertainty. Supplementary different qualities of service are available for the lower level tasks (especially on the video recognition).

State uncertainly are typically handled by Partially Observable MDP (see [14] for a recent reference). POMDP, described in Section 3.2, add some complexity to the MDP problem as the belief into the actual state is probabilistic. Our approach uses a prior FMEA analysis to infer a Bayesian Network model for UAV health diagnosis. In such a way, we avoid the additional computational complexity of the POMDP. A diagnosis approach via Kalman filtering and Baysian networks was experimentally explored in [15] although in an offline fixed mission plan version.

Finally, the mission use case is of comparable complexity with the ones considered in typical civilian single UAV applications (see [16] for example).

In this paper, we focus on MDP model to specify the mission and to elaborate secure mission with a complete autonomy of the UAV. The proposed model is a first step to a complete embedded decision-making engine for the mission planning activity. Overall, our contributions with respect to the state of the art are firstly an online fast MDP algorithm for mission planning based on concurrent MDP. Secondly, we propose state uncertainty diagnosis, which relies on an offline FMEA analysis and online Bayesian Network decision that feeds the decision-making process.

## 3. Decision Model

### 3.1. MDP Model for Mission Specification

#### 3.1.1. Fundamental Model

An MDP is a probabilistic model for decision-making based on Markov chains, which consists of finding the action to execute to switch from state S(i) to state S(i+1) in order to maximize a reward function. The MDP is a quintuplet <S,A,T,R> [17] where:

- *S*: represents the set of system states.
- *A*: represents the set of possible actions.
- *T*: is the transition function defined on S×A×S. Given the current system state *S(i)* and the action *A*, the probability of the next system state being *S(i + 1)* is P[S(i+1)|S(i),A(i)].
- *R*: is the reward function defined on S×A. It indicates the reward obtained when the action *A* is chosen from the current state S(i).

**Figure 1:**
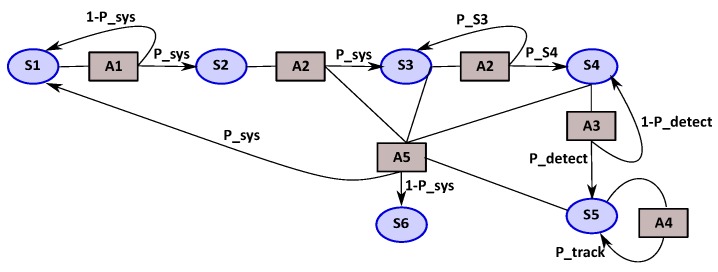
Markov Decision Process for tracking mission.

We illustrate the MDP model in Figure 1 with a classical tracking mission. In this mission, the UAV flies from the starting point to the target area by following a given trajectory. Once at the destination area, the UAV is hovering in order to detect the target and to track it.

1. *System states (S)*: refer to the mission states, which represent the different waypoints covered during the mission. These states are observable by GPS for waypoints states and by applications metrics for the detection and tracking target state:
  - *S1*: ground waypoint (starting point).
  - *S2*: first waypoint in flight.
  - *S3*: set of waypoints between the starting point and the tracking area (arrival area).
  - *S4*: represents the detection target state.
  - *S5*: represents the tracking target state.
  - *S6*: is the landing state.
2. *Action set (A)*: represents the different actions that will be executed to ensure the mission:
  - *A1*: is the take-off action.
  - *A2*: represents the “follow trajectory” action.
  - *A3*: indicates the action to detect the target.
  - *A4*: indicates the action to track the target.
  - *A5*: is the action to return back to the base.
3. *Transition functions (T)*: use the following probabilities:
  - *P_sys*: is the good health probability of the system including battery level estimation.
  - *P_S3*: probability to stay in S3 and to follow the waypoints of the trajectory.
  - *P_S4*: probability to leave S3; it means that the next waypoint is the final of the intermediate waypoints.
  - *P_detect*: is the probability of the target be detected.
  - *P_track*: is the probability of having a good tracking, in terms of QoS.
  In our approach, these probabilities are computed by the dedicated BN as detailed in Section 4.
4. *Reward functions (R)*: the rewards are fixed according to the priority of the actions during the mission and oriented by the probability values.

#### 3.1.2. Cost Criteria and Solutions

The decision-making in MDP models is expressed as a policy. A policy π is a rule used to choose control actions. Although the rule definition may be arbitrary, most approaches limit the choice of control actions as state-only dependent, such as when, in state S(i), the choice of the action A(i) depends only on the state S(i). Solving an MDP means choosing the optimal control actions that will maximize a reward or, equivalently, minimizes a cost criterion. Several cost criteria are classically used. Typical reward (or costs) criteria include instantaneous or average discounted and undiscounted costs over finite or infinite time horizons in continuous or discrete time. In our setup, we are concerned by the undiscounted cost on finite horizon in discrete time:

(1) $$V_{\pi }\left(S\left(i\right)\right)=E_{\pi }\left[\sum_{k=0}^{N}R(S\left(k\right),A\left(k\right)\right],$$

where $V_{\pi }\left(S\left(i\right)\right)$ is the *N*-step cost under policy π when the initial state is S(i), *E* is the expectation operator and R(S(k),A(k)) is the cost of the action A(k) in state S(k).

Classical solutions (i.e., finding optimal policy $\pi^{*}$ which maximizes the reward—or equivalently minimizes the cost—(1)) are obtained by dynamic programming. Two main algorithms are classically used: value-iteration and policy iteration. Value iteration algorithms are based on Bellman equations in a recursive form expressing the reward (cost) in a state as an instantaneous reward plus a residual cost-to-go. For example, from the undiscounted cost on finite horizon in discrete time (1), one can write the recursive Bellman equation:

(2) $$\begin{array}{ccc} V(S(k)) & = & R(S(k),A(k))\\ & + & \sum_{j=0}^{N}\left(P(S(j),S(k),A(k))V(S(j))\right), \end{array}$$

where P(S(j),S(k),A(k) is the transition probability from S(k) to S(j) under action A(k) and V(S(j)) is the residual cost to go after performing A(k). The value-iteration algorithm will build the optimal policy while recursively computing the state rewards. At each step, the best action is chosen in order to maximize the reward in (2) according to:

(3) $$\begin{array}{ccc} V^{*}\left(S\left(k\right)\right) & = & max_{A}R\left(S\left(k\right),A\left(k\right)\right)\\ & + & \sum_{j=0}^{N}\left(P\left(S\left(j\right),S\left(k\right),A\left(k\right)\right)V^{*}\left(S\left(j\right)\right)\right), \end{array}$$

where V∗ is the maximized reward.

In contrast, the policy-iteration algorithm will choose an initial policy and then solve the linear system:

(4) $$\begin{array}{ccc} V_{\pi }\left(S\left(k\right)\right) & = & R(S(k),A(k))\\ & + & \sum_{j=0}^{N}\left(P\left(S\left(j\right),S\left(k\right),A\left(k\right)\right)V_{\pi }\left(S\left(j\right)\right)\right), \end{array}$$

and finally compute the optimal policy for each state as

(5) $$\begin{array}{ccc} \pi^{*}\left(S\left(k\right)\right) & = & {\text{arg\_max}}_{A}\left(R(S(k),A(k))\right.\\ & + & \sum_{j=0}^{N}\left(P\left(S\left(j\right),S\left(k\right),A\left(k\right)\right)V_{\pi }\left(S\left(j\right)\right)\right)). \end{array}$$

The complexity of both algorithms is important. Per iterations, the value-iteration performs at worse in $O(|A||S{|}^{2})$ and policy-iteration in $O(|A||S{|}^{2}+|S{|}^{3})$. The complexity depends on the sparsity of the transition matrix. In the finite horizon undiscounted case, there is no notable difference between the number of iterations performed by value-iteration and policy-iteration; therefore, the value-iteration will be preferred. In general, policy-iteration converges faster while value-iteration may need an exponential number of iterations in the undiscounted infinite horizon case. Some intermediate algorithms (like modified policy iteration) attempts to make a compromise between the number of iterations and their complexity.

### 3.2. Partially Observable Markov Decision Processes

Several versions of MDP problem were developed. The Partially Observable Markov Decision Processes (POMDP) are of particular interest for the UAV mission planning. In POMDP methods, the state observation is uncertain, and so a probability distribution is associated with each state. Two new parameters are added to the Markov model: a set of observations Ω and a set of observation probabilities $P(o|S^{\prime }\left(k\right),A\left(k\right))$ where *o* is the observation when the process reaches state $S^{\prime }\left(k\right)$ after action A(k). There are two main ways to tackle with POMPD: either working on the observed process (i.e., the observations *o* are the states of a new decision process) or mapping each observation *o* to a state S(k) based on a belief (for example, taking the most probable state, which may correspond to a given observation). Working on the observation space will usually arise in intractable non-Markovian processes. Mapping observation to the most probable states allow for treating POMDP as MDP although the most probable state to map to is not always easy to find (e.g., in the trivial case when two states correspond to the same observation with probability 0.5). For the UAV mission planning applications, POMDP are of interest while the internal state of the drone is usually obtained by a diagnosis algorithm. Examples of UAV related applications of POMDP may be found, for instance, in [18,19,20].

### 3.3. Conflicting Concurrent MDPs

Classical MDP frameworks assume that a single action may be executed at a time. Extended frameworks allows several actions chosen from a non-conflicting action set to be executed at the same time [21,22]. When the system runs with limited resources, conflicts may arise between concurrent actions. The setup is investigated in [23]. The proposed framework is composed by several concurrent MDP sharing limited resources. Two types of conflicts may arise: resource conflicts (when the limited amount of resources will limit the number of simultaneous actions) and behavioral conflicts (when executing optimal actions in two different concurrent MDP will result in a global non-optimal or even undesirable behavior). For solving the resource conflicts in [23], a new MDP is constructed over the set of conflicting actions. A policy-iteration algorithm is used to select the maximal reward sub-set of non-conflicting actions. The behavior conflicts are addressed with a set of application specific rules that restricts the execution of some actions if other actions are already in execution. In our setup, we consider different behavior conflicts and resource conflicts. The solving of the first one is based on expert decisions about action priorities and are captured offline. The second one also relies on actions priorities decided by experts offline but also on possible application QoS degradation computed online according to resource availability.

### 3.4. Positioning

Many previous works consider the uncertainties of the environment to manage a UAV mission. POMDP is, for instance, an efficient model that is used to solve the problem of mission management by taking the efficient decision during the mission. A POMDP approach includes everything in a single model, such as the decision related to motion control, security (obstacle avoidance) and all mission phases (e.g., tracking based on detection event), essentially in the case of localization and orientation [24,25,26]. This approach offers good results; however, it is neither tractable and nor scalable if we include all decision-making categories including algorithm versions and optimization of computing resources.

Our approach aims separation of concerns. The first level deals with mission phases (navigation, tracking, obstacle detection, etc.) and can be specified by the mission designer. The second level includes different modules of diagnosis based on BN; they are organized in three categories: health of the system (resources, battery, etc.), the health (QoS) of applications and the health of sensors.

**Figure 2:**
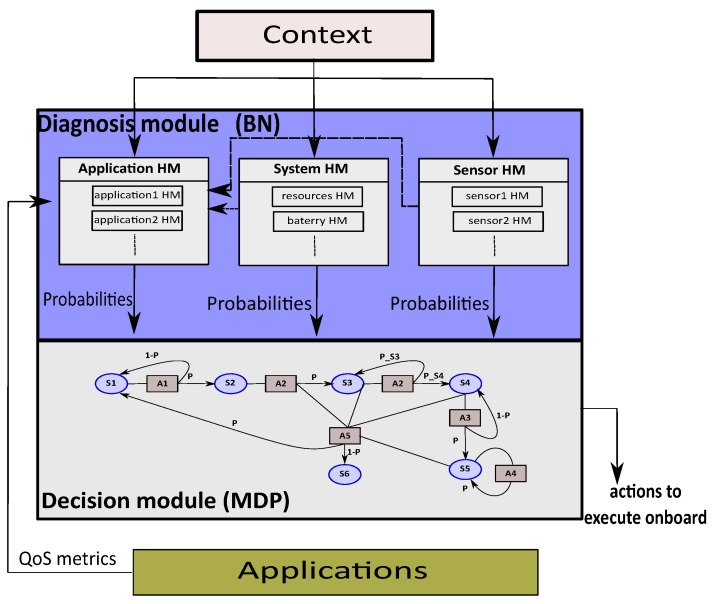
BFM (Bayesian network from FMEA analysis used by MDP) model architecture for mission management.

Figure 2 shows the architecture of the proposed approach. Our model separates and interconnects the diagnosis module and the decision module. The diagnosis module computes the health state in terms of probabilities of the different Hardware (HW)/Software (SW) components of the system (sensors, system and applications) considering the context hazards and the internal event for the applications. Different types of Health Management (HM) are then available to feed the decision module (HM for applications, HM for sensors, HM for system). The “applications HM” contains multiples HM, one for each application that can run on the embedded system (e.g., tracking, navigation, obstacle avoidance, etc.). The “sensor HM” also contains different HMs for the different sensor or set of sensors (e.g., GPS, camera, etc.). Finally, the “system HM” includes the HM of the other components of the system as resources (i.e., CPU load, etc.), battery, etc. The sensor health and system health can impact the QoS of the application. The different HM modules are elaborated using a BN model.

The decision module is based on an MDP model to get the list of actions to execute. The MDP model takes as inputs the probabilities computed by the different HM of diagnosis module. These probabilities are carried by the transition functions of the MDP.

Self-adaptative SoC are implemented with reconfigurable architectures and adapt the HW/SW configurations according to applications demands (e.g., [27]). However, they usually don’t decide the applications, the algorithm to run neither the QoS nor the timing constraints. Our solution is complementary to existing work since it lays on the top of self-adaptive architectures. Moreover, it is designed in such a way that the diagnosis module can be updated at run-time with observed execution times that are used to feed the probability models.

## 4. BFM Model

### 4.1. Health Management by Means of Bayesian Networks

BN is a probabilistic model used to evaluate the health status of the system by detecting errors that can be observed in a certain context. The nodes of a BN represent random variables, and the edge between two nodes represent a conditional dependency. Each node of the network has a probability table (CPT), which indicates the conditional probabilities.

**Figure 3:**
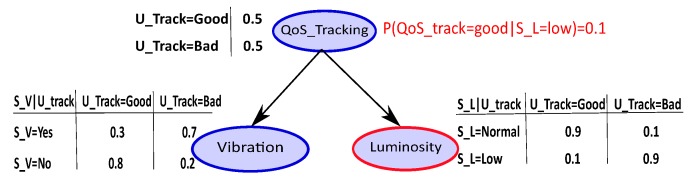
Simple example of Bayesian Network.

A simple example of BN is given in Figure 3. The node Tracking (U_Track) represents the QoS tracking (good or bad) considering the context. The Vibration (S_V) and Luminosity (S_L) nodes represent the sensor’s observations of whether the QoS tracking is good or not. The CPT values are fixed on the basis of knowledge of system’s components and their interactions. In practice, the CPT can be updated online by means of learning techniques applied to BN parameters [28].

Let’s consider a simple example in Figure 3, we observe a context with “luminosity = low” (put evidence on the luminosity node); then, the probability obtained of the QoS tracking is “good” at 10%. The computation of this probability is given by inference.

The BN model for diagnosis takes into account the uncertainty of the mission context. Thus, the elaboration of the BN model is not an easy task. Thus, we introduce the knowledge and experience of experts by means of FMEA tables. Such tables contain the main errors context with the possible monitoring and solutions; an example is given hereafter.

### 4.2. BN for QoS Tuning

During UAV missions, the evaluation of health status of the components of the system (i.e., sensors, actuators, etc.) is necessary [29]. On the other hand, the QoS status of the applications which are executed on board, such as tracking application, can also be monitored to ensure mission success.

In this section, we propose that FMEA tables can be used for the tracking task based on the context monitoring. The FMEA table contains the possible errors that can decrease the QoS of the tracking application considering the context. Depending on environment factors, different parameters or versions of the tracking algorithm can be considered to achieve a good QoS, as shown in Table 1.

**Figure 4:**
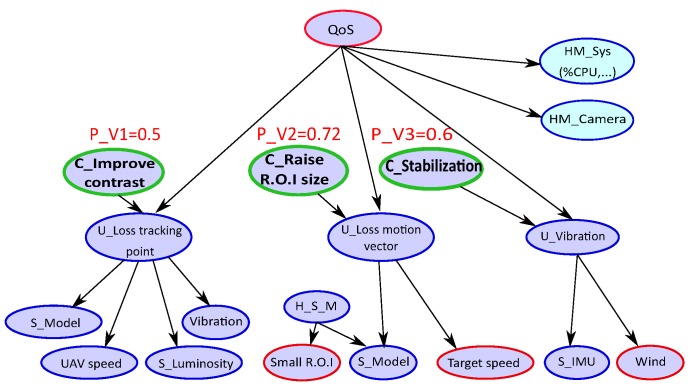
BN for tracking application from Failure Mode Effects Analysis.

Figure 4 shows the translation of the FMEA Table 1 of the tracking application into a BN model. The root node represents the QoS of the tracking application to maintain when an error context occurs. The **U_nodes** indicate the unobservable state of the errors of vibrations (**U_Vibration**), motion vector (**U_Loss motion vector**) and error of point tracking (**U_Loss point tracking**). These types of errors are monitored by physical (sensors) or software measurements denoted by **S_nodes** as (**S_IMU**, **S_Model**, …) and appearances context. Monitor nodes can also have a health status indicated by the **H_S_node** as (**H_S_M**) in a certain appearances context **A_H_nodes** (e.g., **Small Region Of Interest.**). The **C_nodes** represent the solutions that can correct the observed type error (e.g., **C_Stabilization** in the case of vibrations error), and ensure maintaining the expected application QoS level.

Now, we present an example of how to use the BN model. Figure 4 shows the computation of probabilities associated with solution nodes (green nodes). As mentioned previously, the nodes represent random variables. Each node has two states except the QoS node which has three states (high, medium, low).

In this example, we observe a context with wind and target speed and we fix the probability (evidence) of QoS to be in the state “high”. The wind introduces some vibrations and tracking error occurs. The target speedup can also lead to an incorrect motion vector estimation if the R.O.I. is too small regarding the target speed. These observations are reported in the BN by providing evidence on these context nodes, represented by the red nodes in Figure 4. We obtain a probability of 60% to activate the stabilization and 72% to activate new tracking version by increasing the window (R.O.I.) size. In this example, the efficient solution is to increase the R.O.I. size.

As shown in Figure 2, the diagnosis module of the BFM model is composed of different categories of HM (HM applications, HM sensors and HM System) so that the health status of different components (tracking application, GPS sensor, battery, etc.) can be specified and evaluated independently. Typically, designers can consider one BN per application in the applications HM category (BN-tracking, BN-stabilization, etc.). The same principle is applied to the decision module. The mission can be specified by multiple MDPs, each mission phase can be represented by an MDP/BFM and security, navigation and mission-specific applications will be specified with distinct MDP. This scalable approach allows for taking advantage of parallel architectures such as hybrid CPU/FPGA architectures for instance.

### 4.3. Resource-Aware HM

The performance of an application depends on its implementation. For instance, to be able to achieve a 150 FPS (frames per second) for the tracking application, we need a parallel version implemented with FPGA hardware resources. For each embedded application, different variants can be defined to fit the performance needs. For the tracking application, we can have, for instance, a fast version (version *A*) of 150 FPS that runs on FPGA using all resources, a slow one (version *C* at 5 FPS) that runs on CPU only, and an intermediate one (version *B* running at 50 FPS) that runs a the CPU with a coprocessor implemented on the FPGA.

In this section, we show how we can enrich the HM to take into account the resources constraints of the system and the performance constraints.

#### 4.3.1. Resource Constraints

First, the resource constraints are introduced into the HM as BN nodes. The probabilities of these nodes correspond to the load of the device chosen to execute the embedded applications. A large panel of computing resources can be considered such as GPU, multi-cores, FPGA, or heterogeneous architectures such as FPGA-SoC. The resource constraints can be roughly expressed in terms of number of cores for multi-core CPU, number of workgroups for a GPU and in terms of number of tiles (predefined dynamically reconfigurable area) for the FPGA. In a case of hybrid devices like FPGA-SoC (e.g., Altera Cyclone V, Xilinx Zynq), we define the metric $U_{App_{i}}$ corresponding to the resource occupancy for one application $App_{i}$, where the fractional part of $U_{App_{i}}$ represents the CPU load and where the whole part of $U_{App_{i}}$ represents the FPGA load in terms of tiles.

**Figure 5:**
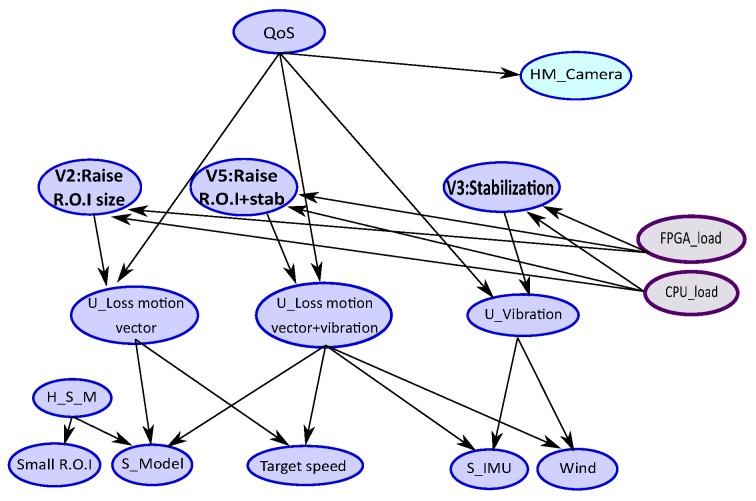
Resource-aware Health Management (HM).

These two loads (FPGA and CPU) can be separately modeled with two different nodes in the HM whose number of states depends on the parallel grain chosen for each support. For instance, in case of the FPGA, the number of states can be associated with the number of tiles; and, in a case of CPU, the number of states depends on the different interesting thresholds for the CPU use (typically 20%, 40%, 60%, 80%). Figure 5 illustrates the insertion of this resource constraint into the resource nodes. In this example, we consider a FPGA-SoC device and two resource nodes are inserted. Four tiles are under consideration for the FPGA, and two thresholds (%30 and %80) for the CPU. The values of these nodes correspond to information monitored by the system and, based on this information, we estimate the capability of the chosen version to be executed by the device. We estimate two possible states ToActivate or ToDesactivate of the version. This estimation is elaborated through the conditional probability tables of the solution nodes of the HM related to the resources nodes. If we consider the version *A* of the tracking, we need to use all the resources of the FPGA, so the activation of this version is possible only if the monitor of the system indicates a total availability of the FPGA.

#### 4.3.2. Performance Constraints

The implementation of the solution nodes to be considered depends on the performance required. The choice of the most adequate implementation of the version depends on the relative speed between the drone speed and the tracked target speed for the tracking application. The ’just enough’ management proposes to not choose the fastest version but the fast enough in order to use only the necessary resources to satisfy the performance constraints.

**Figure 6:**
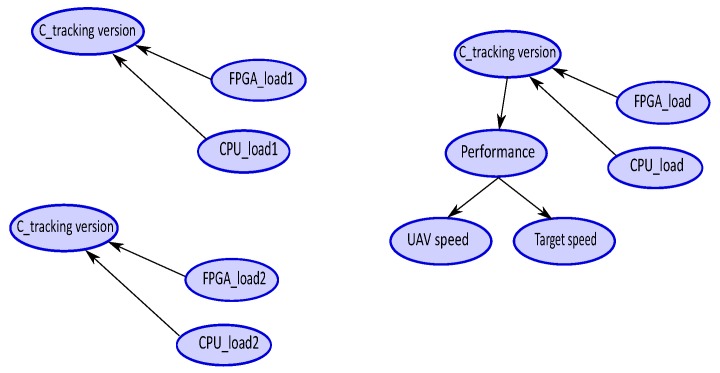
Resource adaptation for performance.

The performance constraints can be introduced in two ways, as shown in Figure 6:

- by modifying the probabilities tables of the solution nodes (${\text{C\_tracking}}_{v}ersion$) or
- by adding new nodes to estimate the performance (Performance node) which are connected to the solution nodes

The two proposals have obtained the same objective; they modify the resource estimates by taking into account the appropriate implementation version. In the first case, the new probability tables are downloaded and, in the second case, based on the monitoring of the speed of the drone and of the speed of the target, we use the inference computation of the BN to drive these modifications.

### 4.4. Example of BFM Model for the Tracking Application

In this section, we consider a UAV mission of tracking where the UAV flies from the start point to arrival area by following a given trajectory. When it reaches the arrival area, the UAV is hovering over this area with the aim to detect a target and track it.

During the mission, different errors can occur due to hazards of the mission. In this example, we focus on the tracking phase of the mission and we consider five possible variations from the nominal version (**V0**) for the tracking application.

- **Version 1**: Histogram-based equalization is added to the reference tracking application to improve the contrast of the image.
- **Version 2**: The tracking version considers a bigger size for the image (resize the R.O.I.—Region of Interest).
- **Version 3**: The tracking evokes extra image stabilization.
- **Version 4**: Tracking version 1 (histogram) with stabilization.
- **Version 5**: Tracking version 2 (R.O.I. resizing) with extra stabilization.

**Figure 7:**
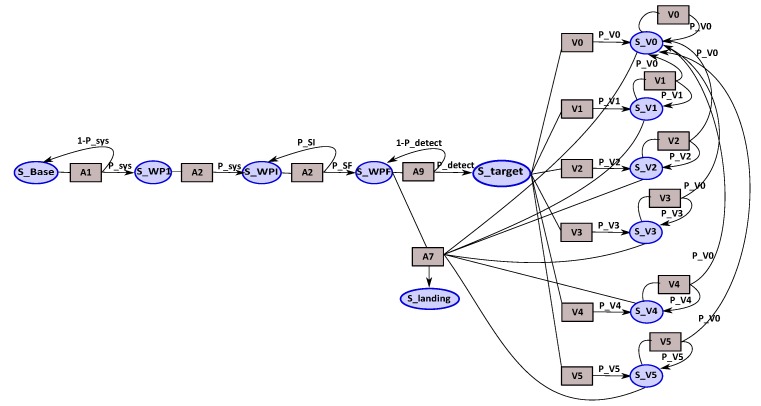
MDPs for tracking version mission.

The adaptive tracking mission is modeled using the BFM model. The nodes of the MDP represent the different states of the mission, and the squares represent the possibles actions (including the application versions) that can be chosen as shown in Figure 7.

With the aim to take an efficient decision related on MDP, we need to specify the MDP transition probabilities values of the different versions of the tracking application. These probability values are computed by the BN diagnosis modules as shown in Section 4.3. The reward values are oriented by the versions’ probability values, if the probability of Version 1, for example, is higher than the other versions, then its reward is the higher one.

## 5. Monolithic BFM Model for UAV Mission

In this section, we describe the global MDP that represents the whole UAV mission. We consider the tracking mission where the UAV follows a predefined trajectory (set of WPs); once arriving at the search area, the UAV tracks the potential target when it is detected. To ensure this mission, we need to execute different actions—some of these actions for navigation (follow a path) and safety (obstacle detection) and other actions to track the target.

1. Set of actions:
  - A1: take-off.
  - A2: follow trajectory (set of WPs).
  - A3: obstacle avoidance.
  - A4: obstacle detection by sensor fusion (IR, Us).
  - A5: “T” zone search for landing.
  - A6: emergency area search for landing.
  - A7: landing action using the camera servo control.
  - A8: replanning and landing action.
  - A9: target detection.
  - A10: back to base.
  - A11: obstacle detection by LIDAR.
  - A12: nominal version of tracking application (V0).
  - A13: version V1 of tracking (V0 + Histogram).
  - A14: version V2 of tracking (V0 + R.O.I. resizing).
  - A15: version V3 of tracking (V0 + Stabilization).
  - A16: version V4 of tracking (V1 + Stabilization).
  - A17: version V5 of tracking (V2 + Stabilization).
  We also define the set of states that represent the different states of the mission. These states are:
2. Set of states:
  - S_base: departure state from base.
  - S_WP1: first waypoint of trajectory.
  - S_WPi: intermediate waypoints of trajectory.
  - S_WPf: final waypoint of trajectory corresponding to the tracking area.
  - S_obstacle_detected: an obstacle is detected by LIDAR or sensor fusion.
  - S_fusion: indicate the obstacle detection state using fusion application.
  - S_WP_avoid: set of waypoint resulting by obstacle avoidance.
  - S_“T”_zone_Ok: “T” zone is detected for landing.
  - S_“T”_zone_Nok: “T” zone not detected.
  - S_zone_Ok: emergency area is found to land.
  - S_zone_Nok: emergency area is not found.
  - S_landing: landing state.
  - S_target: indicate the target detection state.
  - S_Vi: represents all states corresponding to the different versions of the tracking application, for i = 0...5.

**Figure 8:**
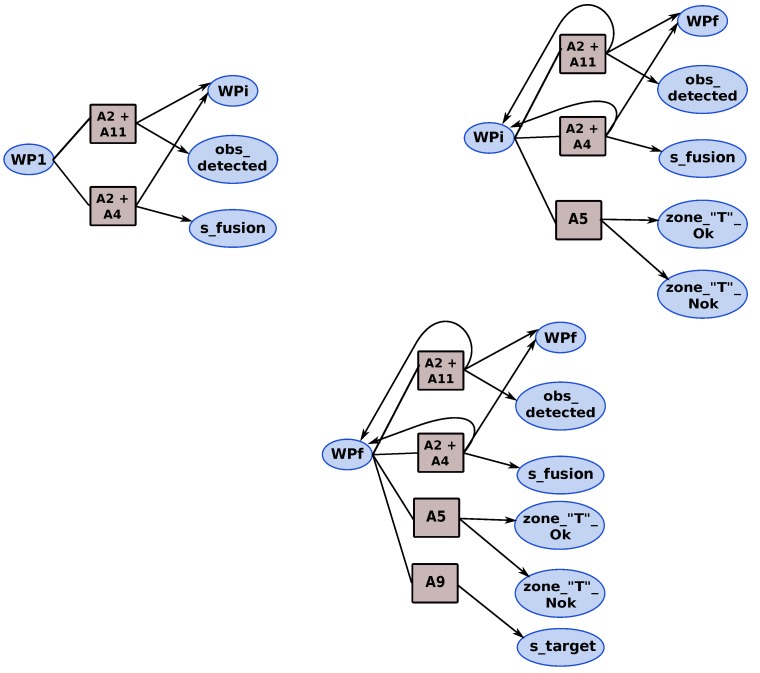
Fragment of Markov Decision Process from Way Points states of tracking mission.

Now, the different states and actions of the mission are defined; we can build the MDP that represents the tracking mission by defining all the necessary transitions that permit moving from one state to another by choosing an action Ai. We illustrate the MDP mission by showing some fragment of the MDP. Figure 8 shows the different actions that can be applied from the different waypoint states (WP1, WPi and WPf). The actions A2 + A11 (follow trajectory & obstacle detection by LIDAR) and A2 + A4 (follow trajectory & obstacle detection by sensors fusion) are used to navigate the waypoints of the trajectory; thus, they are executed from the waypoints’ states. According to the mission description, we add the adequate actions. For example, the action A5 (“T” zone search) can be executed from WPi state and WPf state comparatively to the action A9 (target detection) which can only be activated when we reach the final search area (WPf). When the potential target is detected, the tracking application is activated with the appropriate version according to the context observed.

**Figure 9:**
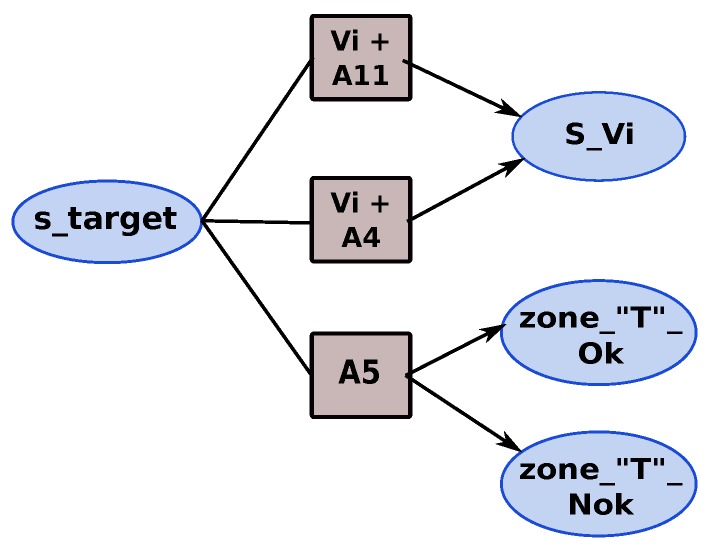
Representation of tracking phase under mission policy.

**Figure 10:**
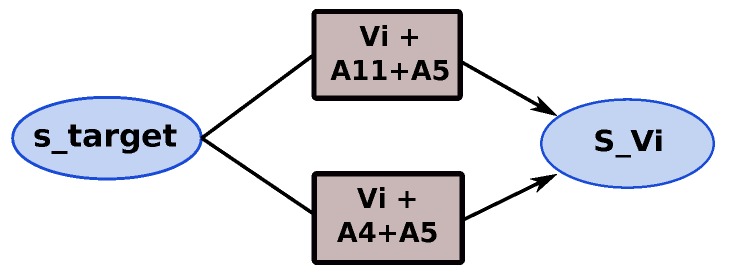
Tracking phase under safety policy.

The obstacle detection (LIDAR & fusion) and area search (“T” zone & free zone) for landing actions are considered as safety actions. The priority given to the obstacle detection action is higher than the priority of search landing area. The objective of our mission is to track the target as long as possible. Thus, two mission policies (strategies) are defined “*safety policy*” and “*mission policy*”. Depending on the mission policy chosen, two representations of the tracking phase of the mission are possible, as shown in Figure 9 and Figure 10. With the mission policy, the tracking application and the obstacle detection are executed in parallel; on the other hand, the tracking, obstacle detection and search landing area applications are executed in parallel in the case of safety policy.

The probability carried by the MDP transitions are produced either by the diagnosis module (sensor HM, system HM, application HM) or by the embedded applications (trust of tracking application, probability of obstacle detection, etc.). The rewards are fixed according to the mission policy.

With the aim to maximize the tracking time of the mission, we solve the tracking mission MDP which has 21 actions and 20 states. Thus, we have 21 transitions matrices where each matrix has a size of 20 × 20 (nb_state × nb_state) and a rewards matrix of 20 × 21 size (nb_state × nb_actions). In the next section, we present the concurrent BFMs approach to reduce the complexity of monolithic BFM.

## 6. Concurrent Versions of BFMs

We propose in this section a scalable methodology to specify a whole UAV mission that incorporates different embedded applications (tracking, sense&avoid, and zone detection for landing). We first express separately the different applications in different BFMs considering the potential sensor failures (sensor HM), system failure (resource HM) and the different alternatives for the embedded applications (application HM) that drive the choice and the number of BFMs. This separation makes the specification tractable, but the use of concurrent MDP require solving possible conflicts if different states are chosen. Our approach takes advantage of the formalisation offered by the BFM model to interact with experts who can specify how to solve the conflict according to a policy choice (e.g., conservative vs. risky) and a context (e.g., safe or dangerous). Finally, we need to solve two kinds of conflicts, behavior conflict and resource conflict, to achieve the global mission.

### 6.1. Separate BFM Specification

For each set of applications, we elaborate a BFM model in order to achieve a tractable and logical specification based on the choice of the different alternatives. The MDP part of each one is built upon observable states and the actions to fix to move from a state to another. An action corresponds either to a drone command as a navigation phase or to an application running on the embedded system.

The MDP are not indepedent and the concurrent execution imposes the following constraints: Here are some constraints:

- All BFMs have the same departure state, the same final state.
- Some common states are required for the synchronization of the BFMs.

To illustrate the concurrent BFM specification, we define three main MDPs (Navigation, Landing and Tracking) that guarantee the success of the tracking mission. The departure state and the final state in each defined MDP are: base state (S_base), landing state (S_landing). The common states between the different MDPs for synchronization are:

- S_WP1: first waypoint of trajectory (in flight).
- S_WPI: intermediate waypoints of trajectory.
- S_WPF: final waypoint of trajectory corresponding to the tracking area.
- S_landing: landing state.

**Figure 11:**
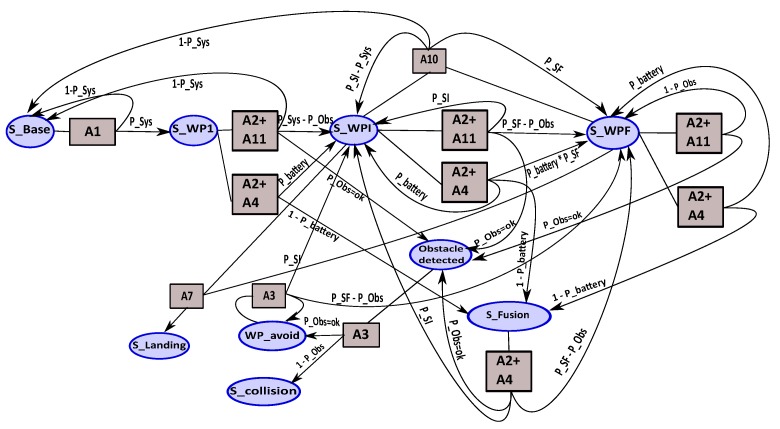
MDP navigation.

The main defined MDPs for tracking mission are presented in the following:

**MDP navigation:** In this MDP, we find the actions of navigation and security. Figure 11 shows the MDP navigation model:

1. navigation states:
  - obstacle_detected: an obstacle is detected by the LIDAR or fusion application.
  - S_fusion: represents the detection obstacle state using a fusion of multiple short/long range infra-red (IR) and ultrasonic (US) sensors.
  - WP_avoid: waypoint resulting by obstacle avoidance.
  - S_collision: collision state with the obstacle.
2. navigation actions:
  - A1: take-off action.
  - A2: follow waypoints of trajectory.
  - A11: obstacle detection using LIDAR.
  - A3: obstacle avoidance action.
  - A4: obstacle detection by sensor fusion (IR, US).
  - A7: landing action.
  - A10: back to base.
3. transition functions:
  - P_sys: probability of good health of the system.
  - P_SI: probability to stay in WPI state.
  - P_SF: probability to leave WPI state for WPF state.
  - P_obs: probability of good obstacle detection.
  - P_battery: estimation of battery level.

**Figure 12:**
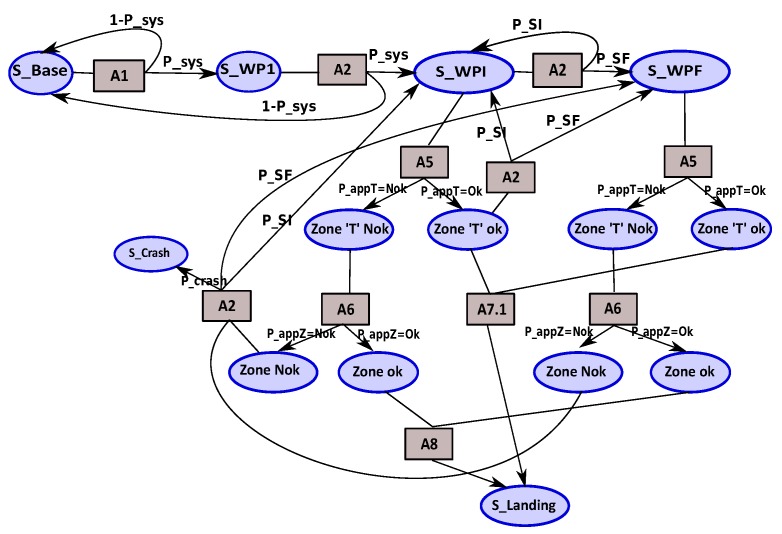
MDP landing.

**MDP landing:** contains the actions required for landing. These actions correspond also to the safety actions. Figure 12 shows the MDP landing model.

1. landing states:
  - Zone_‘T’_Ok: a ‘T’ zone is found.
  - Zone_‘T’_Nok: the ‘T’ zone is not found.
  - Zone_Ok: an emergency area is found to land.
  - Zone_Nok: emergency area is not found.
  - S_crash: crash state.
2. landing actions:
  - A2: correspond to the follow waypoints of trajectory.
  - A5: represent the ‘T’ zone search action.
  - A6: indicate the emergency area search action.
  - A7: landing action using the camera servo control.
  - A8: represent the replanning and landing action.
3. transition functions:
  - P_appT: is the probability that a ‘T’ zone is found.
  - P_appZ: probability that an emergency area for landing is found.
  - P_crash: crash probabilty.

**MDP tracking**: represents the MDP model for the tracking mission with different algorithmic versions of the tracking application. Figure 7 shows this model.

1. tracking states:
  - S_target: represents the target detection state.
  - S_Vi: represents all states corresponding to the different versions of the tracking application cited in Section 4.4.
  The other states of the MDPs (navigation, landing and tracking) correspond to the common states waypoints states) that guarantee synchronization between the different MDPs, departure (base) and final (landing) states.
2. tracking actions:
  - A9: target detection action.
  - V0: nominal version of tracking application.
  - V1: V0 + Histogram.
  - V2: V0 + R.O.I. resizing.
  - V3: V0 + Stabilization.
  - V4: V1 + Stabilization.
  - V5: V2 + Stabilization.
3. transition functions:
  - P_detect: probability of the target be detected.
  - P_Vi: probability of activating a Vi version in a given context by taking into account the resources load and required performance.
  - In addition, there are other transitions that guarantee switching from the current version of tracking application to another tracking version.

All transition functions defined in the three MDPs (navigation, landing and tracking) are computed by: the different HM modules (HM application, HM system and HM sensors); metric applications, etc. The states of the different MDPs are observable by GPS for the waypoints states, battery level monitoring for the S_fusion state of MDP navigation, applications metrics (tracking, sense&avoid, area landing search) for the other states.

### 6.2. Behavior Conflict

Evolving in parallel, the actions decided in each MDP can be incompatible in terms of behavior. We list the different cases and we propose for each incompatibility a solution based on the policy chosen and the expert analysis. This offline approach is tractable since the UAV cases means a limited number of concurrent MDPs and secondly because the number of policies is also limited in practice (e.g., conservative vs. mission first).

In our case study, we have two main potential behavior conflicts corresponding to antagonistic actions, which are listed below:

- Obstacle avoidance, Landing or Return base is antagonistic with Tracking,
- Return base is antagonistic with Landing.

These actions are antagonistic because they require different and incompatible moving reactions of the drone. To solve these conflicts, the expert defines the priority of actions according to the policy chosen. For instance, in a conservative policy (called Safetypolicy), the safety actions are selected first, so Obstacle avoidance is selected instead of Tracking. It can be the opposite with a policy focusing on the mission (Missionpolicy).

In the decision process to solve these conflicts, other criteria can be used such as the risk for the drone, the risk for the environment, the completeness of the mission. In that case, the policy is context dependent and must be solved online after the expert knowledge is captured offline. Then, simple multi-criteria methods can be then applied at runtime as described in [31]. Our diagnosis module provides the sensing of the environment and the health of the system can thus feed such a multi-criteria method.

### 6.3. Resource Conflicts

We focus on the resource conflicts concerning the embedded applications. The same resource is used by different applications at the same time. The resource concerns here the computing resources (CPU, FPGA) but can be extended to other resources as sensors. To solve the resource conflicts of the computing resources, we evaluate the possibility to share the resources evoking different algorithmic versions and their estimated implementations for the required performance.

The different variants for the application are the following:

- Algorithmic variants: different versions for the tracking are available. Priorities between these versions are defined considering the QoS metric associated with the context of the mission provided by the HM applications, as shown in Section 4.2.
- Execution support variants: different supports (HW on FPGA, SW on CPU) can be used for the execution of the algorithmic version leading to various mapping. These mappings are estimated in terms of resource occupancy (FPGA or CPU load) and in terms of performance (number of ms to process one frame for image processing application) as shown in Section 4.3.

The resource-aware HM of each application enables for making the decision between all these variants. In each resource-aware HM, the resources nodes are fixed according to the global observation of the system (monitors are supposed to give this information). The choices of the variants are established in a certain order according to the underlying policy.

In our case study and for the safety policy, we first evaluate the variants of the applications dedicated to safety and then the other actions. Table 2 gives the list of the Application variants under consideration for the case study. For an algorithmic version, we can have three variants for the implementation (CPU or FPGA) that corresponds to a fast (F), slow (S) or medium (M) speed execution. The results are based on HW/SW implementations on a TeraSIC SoCKit board based on a Cyclone V Altera device where Cortex A9 runs Linux with a 925MHz clock frequency. The mission board (SoCKit) communicates with the autopilot which is a standard Pixhawk board The FPGA is divided into four tiles that can be dynamically configured.

## 7. Results and Validation of the BFM Model

### 7.1. Simulation Methodology

The results section is divided into two parts. In the first part, we show the benefit of the QoS tuning for a simple tracking mission. The BFM model is applied to different scenarios of the mission using multiple versions of the tracking application to show the advantage of using the application HM of the BFM model in order to improve the target’s tracking time (see Section 7.2).

In the second part, we present scenarios that illustrate the “just-enough” quality management considering a FPGA-SoC platform for the execution of the embedded applications. In this case, we take into account the resources consumption in addition to the QoS tuning (see Section 7.3). The defined scenarios are experimented under two mission policies (“safety first” and “mission first” policies) to show the policy (result of MDP) impact in both cases.

All of the results are obtained with Matlab simulations we execute the MDP during 100 steps using a finite horizon resolution on the one hand using the monolithic BFM and on the other hand with concurrent BFMs. The probability values used on the MDP transition are given hereafter—note that the probability values of P_Vi (i = 1…5) are obtained using the BN-tracking under vibration context. Thus, these values may change depending on the observed context:

- P_Sys = 0.9,
- P_Obs = 0.5,
- P_Si = 0.6,
- P_Sf = 0.4,
- P_battery = 0.8,
- P_appT = 0.5,
- P_appZ = 0.4,
- P_detectTarget = 0.8,
- P_V1 = 0.37,
- P_V2 = 0.05,
- P_V3 = 0.96,
- P_V4 = 0.31,
- P_V5 = 0.47.

### 7.2. Results for QoS Tuning

In this section, we present the results of our experiments for the QoS tuning for the tracking mission. We used Matlab simulations and applied the BFM model to different scenarios of the tracking mission.

#### 7.2.1. Different Scenarios of the Tracking Mission

We elaborate four different scenarios including different versions of the embedded tracking application to illustrate the benefit of the QoS tuning. These scenarios are as follows:

- **Scenario 1**: nominal case without context errors.
- **Scenario 2**: observation of vibration error through the tracking application BN model
- **Scenario 3**: the target goes faster than the UAV. Thus, there is a high probability of losing the target on the image.
- **Scenario 4**: is an extension of scenario 3 with the presence of strong wind. We observe that the battery consumption decreases more rapidly.

In our case, the estimation of the battery consumption is modeled using a DBN (Dynamic Bayesian Network) model, which is a BN unrolled on two steps. This model computes the probability of the remaining energy taking into account wind, temperature and the new application activated [29].

#### 7.2.2. Benefit Evaluation of QoS Tuning

The MDP of the mission is regularly evaluated and takes as inputs the probabilities provided by the BN diagnosis modules during time. These diagnosis modules are elaborated for the HM of the energy and the HM of the tracking application with the different application versions. We run the MDP during 100 steps with a finite horizon resolution. In a real-life scenario, the steps are event driven. A step is executed when of the CPT content is signficantly changed (e.g., obstacle detection, life time modification, etc.).

We compare the results obtained between the reference mission of tracking and the adaptive mission of tracking (extended version) in terms of success rate which is measured by the number of time steps for the tracking activity, as shown in Table 3. The reference mission is based on the standard MDP implementation described in Figure 1.

The results show that the obtained tracking time varies between the different scenarios presented in the previous section:

- **Scenario 1**: this is the nominal case, the tracking time obtained with the BFM model is similar to the one of reference MDP mission. The tracking is done with success for both, if no hazard appears.
- **Scenario 2**: high vibrations are observed during the tracking phase. We can see that the reference mission of tracking is aborted due to these vibrations. However, with the BFM model, we continue the tracking by activating a new version of tracking application with extra stabilization.
- **Scenario 3**: target speed scenario, the target is temporarily lost. With the BFM model, this problem is corrected by resizing the R.O.I. (Region Of Interest) of the tracking. As a result, the tracking time with the new MDP mission is longer than the reference mission.
- **Scenario 4**: is an extension of scenario 3 with wind. The adequate solution (resize the R.O.I. of tracking) is already activated, but extra energy is consumed due to the presence of wind. As a result, the tracking time with the BFM model is still greater than the one of the reference mission.

The experimentation shows that we can extend the tracking time by considering different possible tracking versions that can correct or decrease the tracking error linked to the context.

On the other hand, we also give the execution time to illustrate the variations of resource use. Execution times on the Cortex A9 embedded processor are obtained with the NEON co-processor optimizations. The tracking application is TLD [30] (aka Predator) with two window sizes (small: 320 × 240 and large: 640 × 480). The stabilization and landing area tracking applications are homemade and use OpenCV functions.

As shown in Table 3, the nominal version can reach about 9 fps (103 × 10${}^{6}$ cycles), if it can use all cpu time. In the context of scenario 2, the new tracking version (nominal tracking + stabilization) is slower and needs 292 × 10${}^{6}$ cycles. In the case of the high speed scenario, the tracking version adopted (double R.O.I. size) is obviously slower and requires 264 × 10${}^{6}$ cycles. Thus, all of these application versions require an extra number of resources to run on a CPU. In consequence, if the extra burden overloads the processor, then the expected QoS cannot be delivered. Thus, it must be considered in the global mission management.

As an example of another possible event-based processing load, we consider an important computer-vision task for emergency landing that identifies flat safe areas. This homemade task requires, if the battery level is dangerously lower than expected, additional 364 × 10${}^{6}$ cycles.

### 7.3. Complete Mission and “Just-Enough” Quality Management

In this section, we present the results obtained for QoS and resource occupation (FPGA, CPU) tuning for the tracking mission. The concurrent MDPs (MDP navigation, landing and tracking) are used for this experimentation.

#### 7.3.1. Scenarios Defined for the Mission Tracking

The experimentation are being done on two scenarios to illustrate the policies obtained by the resolution of the three MDPs (navigation, landing and tracking) for the tracking mission:

- **Scenario 1:** observation of vibration error through the tracking application BN (scenario 2 of Section 7.2 and the presence of obstacles.
- **Scenario 2:** we keep the same scenario 3 presented in Section 7.2 (high speed of target) and we add an obstacle into the trajectory.

In these two scenarios, we take into account the resources used and the required performance during the mission.

#### 7.3.2. Results Obtained with the Monolithic and Concurrent BFM Versions

The three main MDPs (navigation, landing and tracking) of concurrent BFMs guaranteeing the tracking mission are regularly evaluated and their inputs (probabilities transitions) are provided by the diagnosis module of the BFM model. We run the MDP in the same condition as previously (100 steps with finite horizon resolution) to evaluate the tracking time of the mission considering the context errors that can appear. In the first part, we experiment with the two defined scenarios (vibrations and high speed target) on the one hand using the monolithic BFM and, on the other hand, with concurrent BFMs under a safety policy for the UAV and under a mission policy. In the second part, we compare the results obtained between both scenarios under both of the policies (safety and mission) in terms of number of time steps for the tracking phase (tracking activity).

Table 4 and Table 5 show the results obtained in the case of vibration scenarios under the two policies (safety and mission) using the monolithic and the concurrent BFMs. With the safety policy, we can see that, in the tracking phase, the actions of obstacle detection and landing zone search (‘T’ zone and emergency area) have priority over tracking action. When insufficient resources are monitored, the current version of tracking (V3) is degraded to (V1) because V1 takes less resources than V3.

With the mission policy, the priority is given to tracking action but keeping a minimum of safety. Thus, when insufficient resource is monitored, instead of degrading the current version of tracking (V3) application, we disable the landing zone search action and keep the obstacle detection action for minimum safety.

Table 6 and Table 7 show the results obtained in the case of a high speed target scenario with the two versions of BFM model (monolithic and concurrent). The appropriate version of tracking is V2 (resizing R.O.I.). If an insufficient resources event is detected, in case of safety policy, the current version of tracking (V2) is degraded to a new version that is V5; and we maintain the safety actions to activate (obstacle detection and landing zone search). With the mission policy, we detect that the target is moving faster than previously. Thus, we take the fast version of tracking (V2) to guarantee the tracking activity. These results indicate that we obtain the same decision (policy) using monolithic BFM or concurrent BFM.

The resources column of these tables of results represent the estimation of time execution of the set of actions that are executed simultaneously.

Table 8 indicates the results in terms of tracking activity time with both scenarios (vibrations and high speed target). The results show that the tracking mission lasts longer in the case of mission policy than in the case of safety policy. With the mission policy, the tracking application has priority over the other actions like ‘T’ zone search action or emergency area search. Comparatively to the results shown in Table 3, we can remark that the tracking time of the resource aware versions increases compared to the reference mission.

#### 7.3.3. Comparison between the Monolithic and the Concurrent BFM Versions

In this section, we present the comparison between the monolithic and concurrent BFM versions in terms of mission specification, complexity and execution time on CPU. In the first part of our work, we specified and described the tracking mission using a monolithic BFM which is not an easy task because it is necessary to consider all the cases (transitions) of the BFM module that may appear during the mission. With the monolithic BFM, we have 20 states and 21 actions, which means 21 transitions matrices of 20 × 20 size and a reward matrix of 20 × 21 size. The mission description using monolithic BFM is too complex because we need to focus more to define all the states and actions and especially the necessary transitions that allow for moving from one state to another.

To reduce the complexity of the monolithic BFM, we propose the approach of concurrent BFMs. The mission is divided into different phases. In our case, the tracking mission is divided into three BFMs (navigation, landing and tracking); each BFM has respectively nine states for navigation and landing BFMs and 12 states for the tracking BFM; the number of actions varies between 7 and 10 actions. Thus, we can see with the concurrent BFMs that the mission specification is simpler than the monolithic BFM.

The inputs of the different BFMs are probabilities for the transitions and the reward values for each action. The probability values were provided by the diagnosis module of the BFM model. The rewards are fixed according to the mission policy (safety first or mission first) and the actions’ priorities. We solve the tracking mission BFM (monolithic and concurrent) using a policy iteration algorithm, which is an iterative algorithm. The decision is given after policy convergence between two successive iterations. The number of iterations depends on the complexity of the BFM (states and actions number). Table 9 summarizes the the execution time obtained for each BFM and the number of iterations required to converge towards an optimal policy. The results show that the global BFM (monolithic BFM) takes more time to find a policy and needs more iterations comparatively to the concurrent BFMs (navigation, landing and tracking). This is due to the complexity of the BFM in terms of states and number of actions.

We can see that the concurrent BFMs take between 19% and 34% of the execution time relative to the monolithic BFM. The execution time indicates that, if we need to execute the BFM model on FPGA or a multicore processor, the concurrent BFMs are more interesting to use because we can execute them in parallel comparatively to the monolithic BFM.

### 7.4. Summary

In summary, the results of the different experiments show that the BFM model can provide the following two types of benefits:

1. Benefit of the QoS tuning
  - Considering several versions of an embedded application (like tracking application) allows for increasing the duration of the mission (ex: tracking mission).
  - Tuning the QoS of the application allows the system to choose the adequate version of the application to execute considering the observed context.
2. Illustration of “just-enough” quality management considering a FPGA-SoC platform:
  - The simulations also show that the decision made by the BFM model allows the system to adapt to different events (environmental, resource availability, embedded system, etc.).
  - We can see that the policy (set of actions to execute) obtained by the BFM model corresponds to the right plan to execute in each scenario.
  - The simulation also shows the advantage to use concurrent BFMs to reduce the complexity of the mission MDP in terms of mission specification and MDP execution time.

## 8. Conclusions and Perspectives

This paper proposes a new model, named the BFM model, to specify the mission decision that takes into account the environmental context and the capability of the embedded system to perform the required applications for the mission. This model extends the classical MDP model by introducing BN models to interpret the values of the sensors in order to adopt the more appropriate decision concerning the context of the mission. By introducing the resource context of the system, these BNs are also able to select the appropriate version associated with the performance required for the embedded application. This latter one depends on the speed of the drone and the speed of the target in case of a tracking application. The tracking mission is represented first by a monolithic BFM that represents the mission by a complex MDP. In order to facilitate the specification of a complete mission description and reduce the complexity, we propose using concurrent BFMs models and methodology to solve behavior and resource conflicts. We experiment with the concurrent method on a realistic tracking mission using an FPGA-SoC platform to estimate the load and to manage in the most appropriate way the different variants of the embedded applications. Finding the ’just enough’ variants for the embedded applications of the scenarios that we explore gives us a true benefit in terms of mission success expressed with the time spent in tracking.

In the paper, the results are based on real applications’ implementations, but the decision mechanism is simulated. Our ongoing work is the implementation of this approach in a complete real mission with a drone controlled by a FPGA-SoC mission board. The execution time of the different tasks after being estimated by the mission level with concurrent BFM model are validated by the configurable controller [32] that confirms the task management and provides the final schedule of the binary/bitstream on the FPGA-SoC platform for run-time execution. The first future works concern the relevance of the rewards and probabilities of the BFM model, and expect to automatically improve the BN parameters with online learning methods. Secondly, we will consider an HW implementation of the BFM model. Indeed, if the interval between two subsequent steps is not extremely tight, the response time must be critical and the computation of BFM can strongly benefit from HW acceleration.

The study of our approach (BFM model) is done considering a single UAV context, but the multi-UAV case is a promising perspective. The proposed BFM model can actually be extended to a multi-UAV context by representing each UAV using a BFM model and then adding the interconnections between the different UAVs (ex: communication links) With this model, the UAV swarm behavior can be, for instance, implemented with a game-theory based consensus algorithm using the individual MDP solution costs. Then, when a new mission order is issued, UAVs could compute their individual mission costs using the re-planning and reconfiguration MDP model and make decisions based on bets within an auction-like game. 

## Figures and Tables

**Table 1:** Failure Mode Effects Analysis applied to the Tracking Application.

Errors	Possible Monitoring	Appearances Context	Solution (Algorithms)
Vibration	IMU (Inertial Measurement Unit) Vibration sensor	Wind Vibration	Activate the stabilization
Tracking point lost	Model based on: number of features detected (Harris) [30]	Drone speed variations of luminosity	Improve the contrast
Motion vector lost	Model based on: motion vector between 2 images [30]	Target speed Small R.O.I. (Region Of Interest)	Raise the R.O.I. size

**Table 2:** Resources consumption considered for different applications versions for the case study.

Applications	Algorithmic Version	Resources
Tracking	V0: nominal	S(SW: 285 ms) M(HW: 10 ms) F(HW: 7 ms)
	V1: V0 + prefiltering	S(HW: 286 ms) M(SW-HW: 10 + 1 ms) F(HW: 7 ms)
	V2: V0 + R.O.I. resizing	S(SW: 730 ms) M(HW: 10 ms) F(HW: 7 ms)
	V3: V0 + stabilization	S(SW: 540 ms) M(SW-HW: 285 + 10 ms) F(HW: 10 ms)
	V4: V1 + stabilization	S(HW: 540 ms) M(SW-HW: 255 + 10 ms) F(HW: 10 ms)
	V5: V2 + stabilization	S(SW-HW: 410 + 10 ms) M(SW-HW: 30 + 10 ms) F(HW: 10 ms)
Landing area	Zone T search	S(SW: 394 ms) F(HW: 7 ms)
search	Emergency area search	S(SW: 255 ms) F(HW: 7 ms)
Obstacle	Lidar	5 ms
Detection	Fusion	7 ms
Obstacle	V0: right turn	1 ms
Avoidance	V1: path-planning	SW: 1 s
Path-planning [10]	GA version	SW: 1 s

**Table 3:** Comparison of the Reference model and the BFM (Bayesian network from FMEA analysis used by MDP) model for the tracking mission.

Scenario	Tracking Time (nbr Steps)		# Cycles ($10^{6}$)
	Reference	BFM	Tracking version
Nominal	51	51	103 (320 × 240 frame)
Vibrations	40	65	292 (nominal + stabilization)
High Speed	40	72	264 (640 × 480 frame)
Wind	35	56	264

**Table 4:** Policy obtained with scenario 1 (vibrations) under safety policy.

States	Set of Actions	Events	Resources
Base	Tacke-off	No event	-
WayPoint 1	follow trajectory/obstacle detection	No event	5 ms
Intermediate WayPoint	follow trajectory/obstacle detection and search ‘T’ zone	No event	5 + 394 ms
Final WayPoint	follow trajectory/obstacle detection and search ‘T’ zone and target detection	Target detected	5 + 394 + 10 ms
Tracking phase1	V3 and obstacle detection and search ‘T’ zone	Obstacle detected	10 + 5 + 394 ms
	V3 and obstacle avoidance and search ‘T’ zone	Obstacle detected	10 + 1 + 394 ms
Tracking phase2	V3 (Desactivate) and obstacle avoidance and emergency area search	Obstacle detected	1 + 255 ms
	V3 and obstacle detection and emergency area search	Insufficient resources to execute V3	10 + 5 + 255 ms
Tracking phase3	V1 and obstacle detection and emergency area search	No event	10 + 1 ms

**Table 5:** Policy obtained with scenario 1 (vibrations) under mission policy.

States	Set of Actions	Events	Resources
Base	Tacke-off	No event	-
WayPoint 1	Follow trajectory/obstacle detection	No event	5 ms
Intermediate WayPoint	Follow trajectory/obstacle detection and search ‘T’ zone	No event	394 + 5 ms
Final WayPoint	Follow trajectory/obstacle detection and search ‘T’ zone and target detection	No event	5 + 394 + 10 ms
Tracking phase1	V3 and obstacle detection and search ‘T’ zone	Target detected	10 + 5 + 394 ms
	V3 and obstacle avoidance and emergency area search	Obstacle detected	10 + 1 + 255 ms
Tracking phase2	V3 (Desactivate) and obstacle avoidance and emergency area search	Obstacle detected	1 + 7 ms
	V3 and obstacle detection and emergency area search	Insufficient resources to execute V3	10 + 5 + 7 ms
Tracking phase3	V3 and obstacle detection	No event	10 + 5 ms

**Table 6:** Policy obtained with scenario 2 (high speed) under safety policy.

States	Set of Actions	Events	Resources
Base	Tacke-off	No event	-
WayPoint 1	Follow trajectory/obstacle detection	No event	5 ms
Intermediate WayPoint	Follow trajectory/obstacle detection and ‘T’ zone search	No event	5 + 7 ms
Final WayPoint	Follow trajectory/obstacle detection and ‘T’ zone search and target detection	Target detected	5 + 7 + 10 ms
Tracking phase1	V0 and obstacle detection and ‘T’ zone search	High speed target	10 + 5 + 7 ms
	V2 and obstacle detection and ‘T’ zone search	Obstacle detected	730 + 5 + 7 ms
Tracking phase2	V2 (Desactivate) and obstacle avoidance and emergency area search	Obstacle detected	1 + 7 ms
	V2 and obstacle detection and emergency area search	Insufficient resources to execute V2	730 + 5 + 7 ms
Tracking phase3	V5 and obstacle detection and emergency area search	No event	410 + 10 + 5 + 7 ms

**Table 7:** Policy obtained with scenario 2 (high speed) under mission policy.

States	Set of Actions	Events	Resources
Base	Tacke-off	No event	-
WayPoint 1	Follow trajectory/obstacle detection	No event	5 ms
Intermediate WayPoint	Follow trajectory/obstacle detection and ‘T’ zone search	No event	5 + 394 ms
Final WayPoint	Follow trajectory/obstacle detection and ‘T’ zone search and target detection	Target detected	5 + 394 + 10 ms
Tracking phase1	V0 and obstacle detection and ‘T’ zone search	High speed target	10 + 5 + 394 ms
	V2 and obstacle detection and ‘T’ zone search	Obstacle detected	10 + 5 + 394 ms
Tracking phase2	V2 (Desactivate) and obstacle avoidance and emergency area search	Obstacle detected	1 + 255 ms
	V2 and obstacle detection and emergency area search	Higher speed target	7 + 5 + 255 ms
Tracking phase3	V2 (fast version) and obstacle detection	No event	7 + 5 ms

**Table 8:** Duration of the tracking mission.

Scenario	Tracking Time (nbr Steps)	
	Safety policy	Mission policy
Scenario1 (vibrations)	64	68
Scenario2 (high speed target)	71	76

**Table 9:** Software execution time for the different Markov Decision Process on Matlab.

	Number of States	Number of Actions	Execution Time (Millisecond)	Iteration Number
Global MDP	20	21	10.159 ms	5
Navigation MDP	9	7	2.004 ms	1
Landing MDP	9	6	2.781 ms	3
Tracking MDP	12	10	3.537 ms	3
